# Supplementary material for: The interactome of intact mitochondria by cross-linking mass spectrometry provides evidence for coexisting respiratory supercomplexes
Source: Mol Cell Proteomics. 2017 Dec 8;17(2):216–32. doi: 10.1074/mcp.RA117.000470 (PMC5795388; doi:10.1074/mcp.RA117.000470)
Supplement: Supplemental Data [file supp_RA117.000470_133922_0_supp_23978_fzffwf.zip › spectra_annotation/mito_spectra_annotation/493-1-3-1-7-1.pdf]

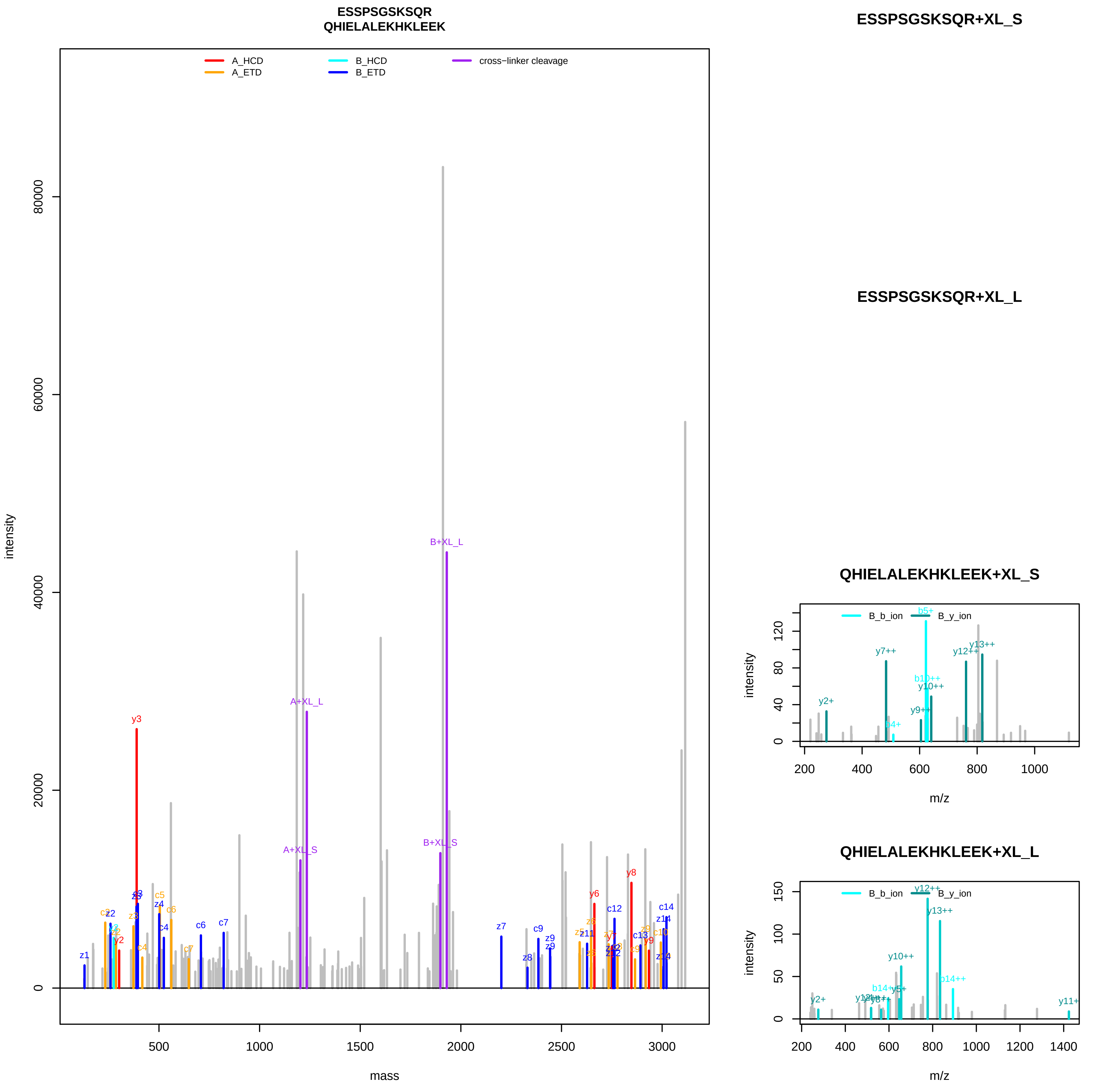
ESSPSGSKSQR
QHIELALEKHKLEEK
A_HCD
A_ETD
B_HCD
B_ETD
cross−linker cleavage
intensity
mass
0
20000
40000
60000
80000
500
1000
1500
2000
2500
3000
y3
A+XL_L
A+XL_S
B+XL_L
B+XL_S
y6
y8
y9
c12
c14
z14
c13
z7
c9
z9
z8
c6
c7
c5
c6
c4
z4
z3
z1
z2
c2
y2
ESSPSGSKSQR+XL_S
ESSPSGSKSQR+XL_L
QHIELALEKHKLEEK+XL_S
B_b_ion
B_y_ion
b5+
y7++
y12++
y13++
b10++
y10++
y9++
y2+
b4+
m/z
200
400
600
800
1000
0
40
80
120
QHIELALEKHKLEEK+XL_L
y12++
y13++
y10++
b14++
y11+
y5+
0
50
100
150
1200
1400
